# Probiotics reduce self-reported symptoms of upper respiratory tract infection in overweight and obese adults: should we be considering probiotics during viral pandemics?

**DOI:** 10.1080/19490976.2021.1900997

**Published:** 2021-03-25

**Authors:** Benjamin H. Mullish, Julian R. Marchesi, Julie A.K. McDonald, Daniel A. Pass, Giulia Masetti, Daryn R. Michael, Sue Plummer, Alison A. Jack, Thomas S. Davies, Timothy R. Hughes, Duolao Wang

**Affiliations:** a Division of Digestive Diseases, Department of Metabolism, Digestion and Reproduction, Faculty of Medicine, Imperial College London, London, UK; b MRC Centre for Molecular Bacteriology and Infection, Imperial College London, London, UK; c School of Biosciences, Cardiff University, Cardiff, UK; d Cultech Limited, Unit 2 Christchurch Road, Baglan Industrial Park, Port Talbot, UK; e Department of Cellular Computational and Integrative Biology, University of Trento, Povo, Italy; f Division of Infection and Immunity, School of Medicine, Cardiff University, Cardiff, UK; g Department of Clinical Sciences, Liverpool School of Tropical Medicine, Liverpool, UK

**Keywords:** Probiotics, gut microbiome, obesity, gut-lung axis, upper respiratory tract infection

## Abstract

Gut microbiome manipulation to alter the gut-lung axis may potentially protect humans against respiratory infections, and clinical trials of probiotics show promise in this regard in healthy adults and children. However, comparable studies are lacking in overweight/obese people, who have increased risks in particular of viral upper respiratory tract infections (URTI). This *Addendum* further analyses our recent placebo-controlled trial of probiotics in overweight/obese people (focused initially on weight loss) to investigate the impact of probiotics upon the occurrence of URTI symptoms. As well as undergoing loss of weight and improvement in certain metabolic parameters, study participants taking probiotics experienced a 27% reduction in URTI symptoms *versus* control, with those ≥45 years or BMI ≥30 kg/m^2^ experiencing greater reductions. This symptom reduction is apparent within 2 weeks of probiotic use. Gut microbiome diversity remained stable throughout the study in probiotic-treated participants. Our data provide support for further trials to assess the potential role of probiotics in preventing viral URTI (and possibly also COVID-19), particularly in overweight/obese people.

## 1. Introduction:

The global burden of disease caused by respiratory tract infections is substantial, representing a significant proportion of physician visits, working days lost through sickness, and healthcare expenditure.^1,2^ Recent large epidemiological studies have observed that being overweight or obese appears to be an independent risk factor that increases an individual’s risk of both upper and lower respiratory tract infections.^3–5^ Comparable findings from the COVID-19 pandemic have shown obese patients having both a high risk of contracting COVID-19 infection, as well as developing more severe disease, including increased mortality.^6^ Whilst lifestyle intervention and bariatric surgery are major current medical options to achieve weight loss and therefore mitigate this risk, any potential additional preventative approaches would evidently be of keen clinical interest.

It is well established that a key function of the microbiota of different mucosal surfaces found in mammals is in colonization resistance, i.e., the prevention of infections either through the direct action of microbiota components upon pathogens or via a number of indirect routes (primarily through interactions with the host immune system^7^). However, a more recent concept has been the recognition of specific interactions and crosstalk between the gut microbiota and the lungs, particularly with regards to respiratory immune and anti-infective responses. This is often described as the ‘gut-lung axis,^8ʹ^ and sometimes referred to as a common mucosal immunological system.^9^ While the mechanisms underpinning the link between obesity and risk of respiratory infections including COVID-19 are incompletely understood, one hypothesis is that obesity-related changes in the gut microbiome composition result in increased intestinal permeability, endotoxemia, and activation of pro-inflammatory host immune responses that increase susceptibility to infection across all mucosal surfaces.^10^ Compared to this, the recognized association between older age and increased risk of URTI may be at least partly explained by the shifts in microbiome composition observed with aging, and associated changes in immune response to infective insults.^11^

By extension, modulation of the gut microbiota as a means of influencing the gut-lung axis has been an area of active investigation, with probiotics being one of the main interventions that has been explored in human trials. A number of different studies have explored the use of probiotics to reduce the risk of respiratory infections, with a focus on viral upper respiratory tract infections (URTI) in particular (90% of URTIs are viral in nature^12^). To date, several systematic reviews and meta-analyses have concluded that probiotics appear to have a positive effect in this regard.^13–15^ However, these systematic reviews have also noted that individual published studies to date are often small, with low-quality data and high risk of heterogeneity, limiting the applicability of their findings to a general population.^13–15^ Furthermore, the majority of these studies have included only healthy children or adults as study participants, with very little focus on groups at elevated risk of viral URTIs, such as overweight and obese adults.

We hypothesized that probiotic use would reduce the risk of symptoms related to URTI in an overweight and obese population, and that this reduction may be related to gut microbiota-mediated mechanisms. In this *Addendum*, we present our additional analysis of a recent double-blind placebo-controlled trial we undertook in an overweight and obese population where probiotics were the intervention, and where the primary endpoint was related to weight loss and lipid metabolism.

## 2. Further analysis of the PROMAGEN study

Our trial examined the impact of probiotics on a healthy, free-living, overweight and obese adult population (PROMAGEN, ISRCTN12562026).^16^ Specifically, this was a double-blind, single-center, placebo-controlled trial that recruited 220 people with BMI 25–34.9 kg/m^2^ (30–65 years of age), with participants randomized to receive either the Lab4P probiotic (50 billion colony-forming units) or a matched placebo daily for 6 months. Lab4P comprised *Lactobacillus acidophilus* CUL60 (NCIMB 30157), *Lactobacillus acidophilus* CUL21 (NCIMB 30156), *Lactobacillus plantarum* CUL66 (NCIMB 30280) *Bifidobacterium bifidum* CUL20 (NCIMB 30153) and *Bifidobacterium animalis* subsp. *lactis* CUL34 (NCIMB 30172). At the end of the study, significant between group decreases were identified in body weight (1.3 kg, *p* < .0001), BMI (0.045 kg/m^2^, *p* < .0001), waist circumference (0.94 cm, *p* < .0001), and waist-to-height ratio (0.006, *p* < .0001), all favoring the probiotic arm. Improvements in small dense LDL-cholesterol, self-perceived quality of life, and the incidence of particular symptoms that may be consistent with URTI were also observed in the probiotic arm relative to participants receiving placebo.^16^ No significant adverse events were observed in association with probiotic use.

**Figure 1.**
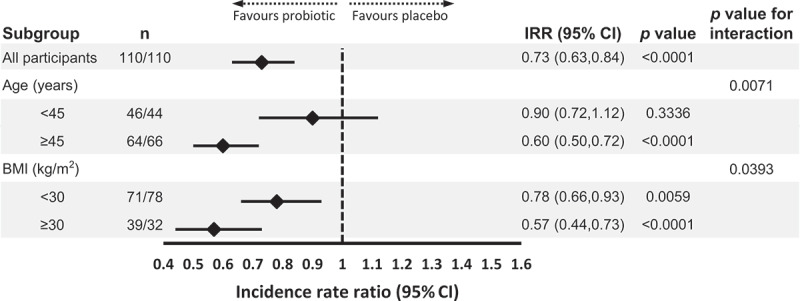
**Overall and subgroup analysis of the impact of probiotic supplementation on the incidence of URTI symptoms**. Statistical analysis was performed using a generalized linear model with Poisson distribution and log link function, data is presented as IRR ± 95% CI (n, number of participants (placebo group/probotic group); IRR, incidence rate ratio; CI, confidence interval)

Study participants also contemporaneously recorded daily symptom diaries during the trial, containing 25 elements related to different organ systems and general health. To explore the potential impact of probiotic use upon URTI symptoms in more detail, we further reviewed frequencies of those symptoms recorded in symptom diaries that could be considered most consistent with URTI. The study was performed over the 6 months from July to January, and as such included a significant period of the conventional peak season for influenza-like illnesses. We selected five key symptomatic indicators of URTI that had been recorded, all of which have been previously included in validated symptom scoring systems for viral respiratory illness/URTI, namely: cough, sore throat, headache, muscle ache and wheeze^17,18^ (‘URTI symptoms’). Further details of the analysis of symptom incidence rate are provided in the **Supplementary Methods**. On review of self-reported URTI symptoms during the study, we observed a significant 27% between-group difference in overall incidence favoring the Lab4P group (Incidence Rate Ratio (IRR): 0.73, 95% confidence intervals (CI): 0.63, 0.84, *p*< .0001 (Figure 1)).

Given the data outlined above regarding age and obesity as risk factors for URTI, we looked at how these factors were related to the recorded URTI symptoms in the PROMAGEN cohort. We analyzed them using a Poisson regression model (Figure 1), which identified significant interactions with symptom incidence between treatment and age (*p*= .0071) and BMI (*p*= .0393, Figure 1). Stratification of the cohort highlighted the greater probiotic impact on incidence rates of URTI symptoms in participants ≥45 years old compared to younger participants (IRR: 0.60, *p*< .0001 *vs* IRR: 0.90, *p*= .3336), and in obese participants compared to those overweight alone (IRR: 0.57, *p*< .0001 *vs* IRR: 0.78, *p*= .0059). Further analysis of additional recorded symptoms of potential relevance to URTIs (but not included as one of our central five symptoms, e.g., sneezing) demonstrated that these occurred at either similar or reduced rates in participants taking probiotics compared to those taking placebo; a pattern was once again observed regarding benefits being generally more prominent in participants ≥45 years of age and with BMI ≥30 kg/m^2^ (**Supplementary Figure S1**).

**Figure 2.**
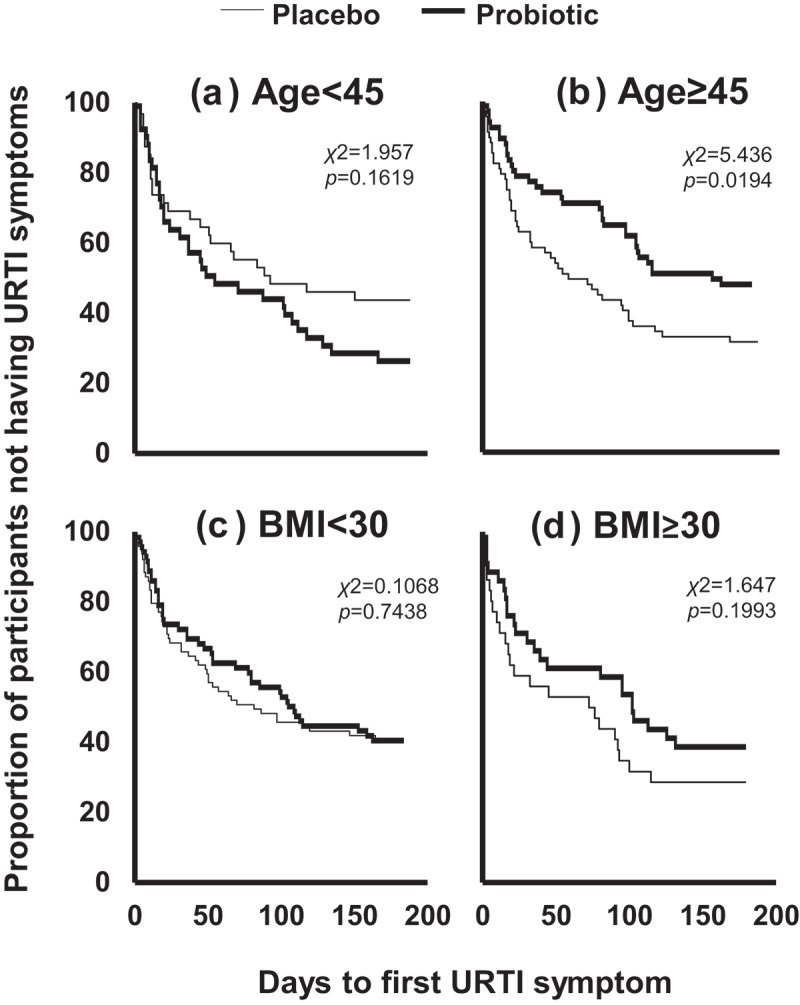
**Impact of probiotic supplementation on time to onset of URTI symptoms during the PROMAGEN study**. Kaplan-meier plots of time to first URTI symptoms in subgroups of participants who were: A) <45 year olds; B) ≥45 year olds; C) overweight (BMI <30 kg/m^2^) and; D) obese (BMI ≥30 kg/m^2^). Statistical analysis was performed using the log-rank Mantel-Cox test

We further explored the dynamics of onset of URTI symptoms. On the first day of the study, participants in both arms of the PROMAGEN study were virtually symptom-free, with only one participant in the ≥45 years old placebo group reporting cough. After 1 to 2 weeks supplementation, there was the clear indication of a divergence between groups in the time taken to record the first symptom favoring the probiotic in the two subgroups, namely those ≥45 years old (Figure 2b) and the obese (Figure 2d), suggestive of a rapid impact of the probiotic upon URTI symptoms. These delays in symptom onset were maintained over the duration of the study in both the older and obese subgroups, and reached significance in the case of the participants ≥45 years old (*χ*2 = 5.436, *p*= .0194, Figure 2d). Collectively, these findings lead us to conclude that Lab4P can act rapidly, particularly in the ’at-risk’ groups of older and obese participants.

**Figure 3.**
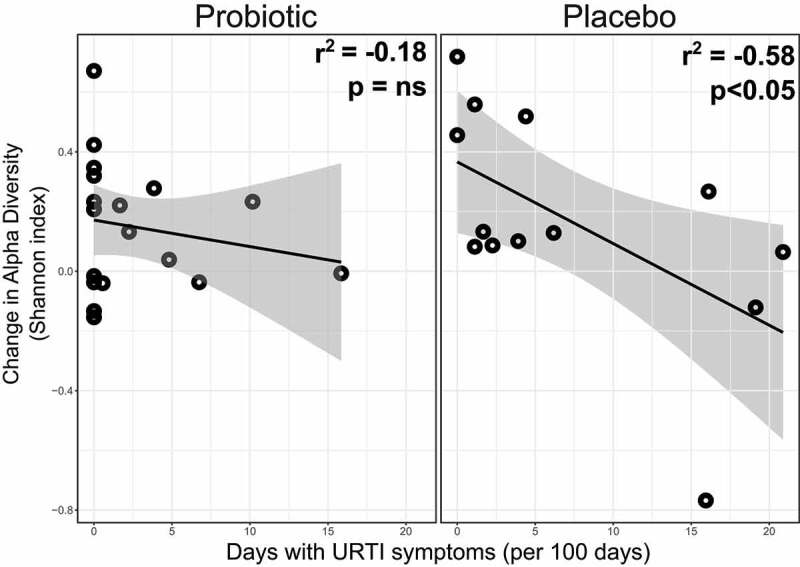
**Scatterplot of relationship between change in Shannon diversity index of the stool microbiota *versus* the number of days with URTI symptoms in participants ≥45 years old given probiotics or placebo**. Shannon diversity was measured in stool samples from study participants ≥45 years old at baseline and at end of the study (six months) using 16S rRNA gene profiling. Statistical analysis was performed using Pearson’s correlation analysis. Probiotic arm: *n = *18; placebo arm: *n*= 13

Additionally, we investigated the associations between the reported URTI symptoms and the gut microbiota profiles of participants ≥45 years old in the study, to particularly explore whether potential URTIs may impact upon gut microbiota composition (Figure 3 and **Supplementary Methods**). Analysis was performed of 16S rRNA gene sequencing of stool samples from study participants ≥45 years old who had given stool samples both at the beginning and end of the study, as previously described.^19^ For placebo-treated participants, a negative correlation was observed between length of time with URTI symptoms and stool microbiota diversity (as measured by Shannon/alpha diversity index; r^2^ = −0.637, *p*< .05). In contrast, the Shannon diversity of the probiotic group was unaltered by the length of exposure of URTI symptoms (r^2^=−0.87, *p*= .473). Of note, only one included probiotic-treated participant had used antibiotics within 3 weeks of the end of the study stool sample (with antibiotics administered for a suspected respiratory tract infection); none of the included placebo-treated participants had used antibiotics within approximately 4 months of the end of the study. Together, these data lead us to suggest that in participants in the study with URTI symptoms, probiotic use may have minimized post-infective changes in gut microbiome composition.

## 3. Discussion:

There is growing evidence supporting the concept that gut commensal bacteria (including those commonly included in probiotics) are able to suppress viruses that enter the host via the upper respiratory tract from causing infection, with research to date focusing particularly upon influenza. Mechanisms by which such bacteria suppress influenza directly include absorptive trapping,^20^ as well as by the production of lipopolysaccharide that binds to and destabilizes the viral structure.^21^ One potential route by which probiotic bacteria may translocate from the gut to the lung (and undertake direct protective functions such as absorptive trapping) is described by the ‘gut-lymph hypothesis’, i.e. gut bacteria within draining chyle from the lower gastrointestinal tract entering the lymphatic system and subsequently the thoracic duct, before traveling directly on to the capillary blood vessels of the lungs.^22^

There are also indirect routes by which the gut microbiota prevents influenza infection, including an influence upon interferon responses in lung stromal cells,^23^ as well as promotion of antigen presenting cell migration and Toll-like receptor-mediated T cell antiviral responses.^24^ Additionally, research into the gut-lung axis in COVID-19 infected patients has shown an altered stool microbiome composition compared to controls.^25,26^ In hospitalized patients with COVID-19, stool levels of a number of gut commensal bacteria (including *Bacteroides dorei* and *Bacteroides ovatus*) were found to correlate inversely with SARS-CoV-2 viral load;^25^ this is of interest, given that such bacteria may downregulate expression in the mouse gut of angiotensin-converting enzyme 2 (ACE2), the receptor used by SARS-CoV-2 to gain entry to the host.^27^ Of particular potential relevance given the data presented in our work here, a further study – longitudinally analyzing the gut microbiome of 100 patients with COVID-19 infection – noted consistent underrepresentation of *Bifidobacteria* in patient stool samples throughout the course of disease.^26^

Furthermore, there is now increased understanding of the mechanisms by which probiotic supplementation of the gut microbiota may directly modulate host immunity in this context, predominantly deriving from mouse models of influenza in which *Bifidobacteria* or *Lactobacillus* supplementation resulted in reduced severity of infection.^28^ There is evidence from such models that *Lactobacillus* supplementation enhances type 1 interferon responses^29^ and increases TNF-α production from nasal lymphocytes in response to respiratory viral infection.^30^ Short-chain fatty acids (SCFAs) – the product of fermentation of non-digestible carbohydrate and amino acids by gut bacteria – appear to be a key group of metabolites linking the gut microbiome to these altered immune responses.^31^ Human trials investigating probiotics for the prevention of viral respiratory infections have shown probiotic-related increases in serum interferon-γ^32^ and Th1 cell activation, increased numbers of T helper cells, T killer cells and monocytes,^33^ as well as changes in NK cell activity.^34^

As discussed above, studies to date evaluating the role of probiotics for the prevention of viral URTI have predominantly focused on healthy adults and children and have tended not to explore outcomes for the overweight and obese population, or older people. There is now recognition of a need for more focused studies within this context, including evaluation of whether probiotics may alter the impact of COVID-19 infection.^35^ Of note, one early study of probiotic supplementation to 28 hospitalized patients already diagnosed with COVID-19 observed eightfold lower rates of respiratory failure in patients receiving probiotics compared to 42 comparator patients receiving medical therapy alone;^36^ however, there are currently no data available to our knowledge which demonstrate efficacy of probiotics for the prevention of COVID-19 infection in humans. The particular novelty of our findings is the recognition that probiotic supplementation may be associated with an overall reduction of URTI symptoms by as much as 27%, with individuals ≥45 years of age and/or with a BMI ≥30 kg/m^2^ deriving the greatest benefit of risk reduction. The recognition that perturbed gut barrier function is associated with vulnerability to both infection and obesity – and that probiotics may be able to improve gut barrier integrity – could be a potential mechanistic link that explains our findings in patients with an unfavorable BMI.^37^ With regards to our findings on the particular benefits of probiotics in older study participants, it is noteworthy that lower levels of gut *Bifidobacteria* have been associated with older age, and *Lactobacillus* and *Bifidobacteria* depletion have also been observed in the gut microbiota of certain patients with respiratory infections, including COVID-19.^38^

The apparent protective impact of the probiotic on gut microbial diversity in study participants who developed URTI symptoms is another finding of interest. While much of the focus to date in gut-lung axis research has been on the impact of the gut microbiome on the lung, the bidirectional nature of this relationship is now increasingly acknowledged. Specifically, changes in gut microbiome composition and functionality post-viral respiratory tract infection that have been observed (even in the absence of detectability of the virus within the gastrointestinal tract) are associated with changes in host immune responses,^39^ and potentially result in increased vulnerability to secondary respiratory infections.^40^ In mouse models of influenza, a reduction in gut *Lactobacillus* has been observed after infection,^41^ while *Bifidobacteria* (including *Bifidobacterium animalis*) were found to be significantly elevated in the gut of surviving mice compared to dead or mock-infected mice.^42^

The major limitation of our study is using self-reported symptoms as a proxy for URTI, without any definitive clinical/laboratory data to confirm true infection; however, the symptoms that we measured show close overlap with those in established symptoms scoring systems for predicting URTIs/viral respiratory illness.^18^ There were significantly fewer of our defined URTI symptoms observed in all participants in the first 3 months of the study (the ‘summer’ months) compared to the last 3 months (‘winter’ months) (*p*= .0108), again supporting the concept that the symptoms which we selected represent winter respiratory viral illness (**Supplementary Figure S2**). Furthermore, since the reporting of URTI symptoms in this study was self-recorded, statistical results may be subject to reporting/recall bias. However, a number of different approaches to symptom recording were taken in order to mitigate the possibility of such bias (**Supplementary Methods 1.1**), and the double-blind nature of the trial means that this is unlikely to have contributed significantly to the between-group differences observed. Whilst we have made some speculation as to the potential applicability of our data to patients with COVID-19 infection, our study was performed before the pandemic occurred, and we did not have any symptom data relating to other potentially important indicators of particular coronavirus infections including COVID-19, such as loss of taste/smell. An additional limitation is that our definition of cough covered any aspect of coughing occurring during the course of the study, and this also may not align with more specific features of URTI (e.g., cough with coryza). Nevertheless, it is clearly of interest that in the PROMAGEN study, even the incidence of cough alone was reduced by ~33% (0.38 per 100 person days in probiotic arm, 0.57 per 100 person days in placebo arm; IRR: 0.67, CI: 0.50, 0.90, *p*= .0073) in participants receiving probiotics compared to those receiving placebo.^16^ As such, one potential area for future exploration may be the potential impact of probiotics upon other human diseases for which cough is a predominant feature, including allergic diseases such as asthma; administration of a *Lactobacillus rhamnosus* resulted in lung protection in a murine allergic respiratory disease model, with the probiotic demonstrated to alter respiratory IL-1β levels, as well as airway total cell counts and lymphocyte counts.^43^

## 4. Conclusions:

In conclusion, our results extend upon existing data regarding the impact of probiotics upon viral URTI, by suggesting that probiotics may have a potential use for reducing URTI symptoms in overweight and obese people, and especially those of older age. Furthermore, the Lab4P probiotic consortium may also have a potential role in stabilizing/preventing changes in gut microbiome composition in response to an URTI. Probiotics are considered overall safe and well tolerated, and relatively limited alternative therapeutic options currently exist for prevention of URTIs (including COVID-19); as such, we feel that a compelling case exists for further randomized studies to prospectively explore the potential impact of probiotics on prevention of respiratory infection in particular for those at higher risk, including obese and older people. Furthermore, there is a need for additional research to better delineate mechanisms by which probiotic bacteria – either directly or via immune modulation – impact upon the gut-lung axis.

## Supplementary Material

Supplemental Material
