# Supplementary material for: Probiotics reduce self-reported symptoms of upper respiratory tract infection in overweight and obese adults: should we be considering probiotics during viral pandemics?
Source: Gut Microbes. 2021 Mar 25;13(1):1900997. doi: 10.1080/19490976.2021.1900997 (PMC8007143; doi:10.1080/19490976.2021.1900997)
Supplement: Supplemental Material [file KGMI_A_1900997_SM3373.docx]

**Probiotics reduce self-reported symptoms of upper respiratory tract infection in overweight and obese adults: should we be considering probiotics during viral pandemics?**

Benjamin H. Mullish, Julian R. Marchesi, Julie A.K. McDonald, Daniel A. Pass, Daryn R. Michael, Sue Plummer, Alison A. Jack, Thomas S. Davies, Timothy R. Hughes, and Duolao Wang

1. **Supplementary Methods:**
   1. **Analysis of symptom incidence rate and time-to-first event:**

The PROMAGEN study design has been described in detail elsewhere^1^. Participants in the study completed daily diaries monitoring URTI symptoms throughout the duration of the 6-month intervention period. These diaries were completed by study participants contemporaneously on a daily basis (rather than retrospectively), and participants were asked to report the presence of any of a broad range of symptoms related to different organ symptoms and general health rather than purely respiratory-related symptoms alone, minimising the risk of response bias. Furthermore, while study participants were aware of weight and metabolism being key endpoints of the study, they were unaware during the study that the impact of probiotics upon URTI symptoms was a specific potential additional area of interest, further reducing the risk of response bias. Symptom record sheets within the diaries were worded in a simple, easy-to-understand way, facilitating accurate symptom reporting for study participants across the range of introspective ability.

Incidence rates (IR) of each symptom or combinations of symptoms were calculated from the daily diaries by dividing the number of symptom episodes (a continuous sequence of symptoms separated from another episode by a minimum of 24 hours) by the number of days the participant was in the study, and are expressed per 100 person days. Incidence rate ratios (IRR, IR active group ÷ IR placebo group) were calculated using a generalised linear model (GLM) with Poisson distribution and log link function. Interactions between treatment and age and BMI were tested using the GLM. Values of *P* were considered statistically significant when less than 0.05. Data analyses were performed using SAS® version 9.4 (SAS Institute Inc., Cary, NC, USA) unless otherwise stated.

Time-to-event for the first symptom in the groups was analysed with the Kaplan-Meier method, and its significance was assessed by the Log-rank Mantel-Cox test (GraphPad Prism, version 8.2.2, CA, USA).

For all statistical analyses, values of *p*<0.05 were considered significant.

- 1. **Statistical analysis of fecal microbiota:**

Fecal samples were collected, stored and processed as previously described^1^. All analysis was performed using Qiime2-2019.10^2^, phyloseq^3^, vegan^4^ and bespoke R code. 128 paired samples (64 participants) were sequenced for 16S rRNA with 63 pairs being retained following quality processing steps using DADA2^5^ and rarefaction at 8000 reads. 29 participants were over 45 years old and used in subsequent analysis (18 Probiotic, 11 Placebo). 4740 abstracted consensus sequences (ASVs) were identified (967 >100 reads (rarefied)). Absolute difference in the Shannon diversity between participants at the beginning and endpoint of the trial were subjected to Pearson’s correlation testing with number of days with URTI symptoms.

1. **Supplementary Figures:**

**A.**

| Subgroup | | | **n** |  | **IRR (95% CI)** | ***p* value** | ***p* value for interaction** |
| --- | --- | --- | --- | --- | --- | --- | --- |
| **Cough** | | |  |  |  |  |  |
|  | All participants | | 110/110 |  | 0.67(0.50,0.90) | 0.0073 |  |
|  | Age (years) | |  |  |  |  | 0.0004 |
|  |  | <45 | 63/70 |  | 1.21(0.77,1.93) | 0.4101 |  |
|  |  | ≥45 | 47/40 |  | 0.40(0.27,0.61) | <.0001 |  |
|  | BMI (kg/m^2^) | |  |  |  |  | 0.0149 |
|  |  | <30 | 71/78 |  | 0.84(0.58,1.23) | 0.3745 |  |
|  |  | ≥30 | 39/32 |  | 0.38(0.23,0.64) | 0.0002 |  |
| **Sore Throat** | | |  |  |  |  |  |
|  | All participants | | 110/110 |  | 0.80(0.55,1.17) | 0.2513 |  |
|  | Age (years) | |  |  |  |  | 0.1156 |
|  |  | <45 | 63/70 |  | 1.21(0.62,2.38) | 0.5711 |  |
|  |  | ≥45 | 47/40 |  | 0.63(0.39,1.01) | 0.0557 |  |
|  | BMI (kg/m^2^) | |  |  |  |  | 0.0017 |
|  |  | <30 | 71/78 |  | 1.22(0.76,1.97) | 0.4048 |  |
|  |  | ≥30 | 39/32 |  | 0.30(0.15,0.63) | 0.0012 |  |
| **Headache** | | |  |  |  |  |  |
|  | All participants | | 110/110 |  | 0.68(0.58,0.81) | <.0001 |  |
|  | Age (years) | |  |  |  |  | 0.2901 |
|  |  | <45 | 63/70 |  | 0.72(0.55,0.95) | 0.0196 |  |
|  |  | ≥45 | 47/40 |  | 0.60(0.47,0.75) | <.0001 |  |
|  | BMI (kg/m^2^) | |  |  |  |  | 0.4468 |
|  |  | <30 | 71/78 |  | 0.67(0.54,0.84) | 0.0005 |  |
|  |  | ≥30 | 39/32 |  | 0.58(0.43,0.79) | 0.0005 |  |
| **Muscle Pain** | | |  |  |  |  |  |
|  | All participants | | 110/110 |  | 0.67(0.48,0.95) | 0.0225 |  |
|  | Age (years) | |  |  |  |  | 0.1494 |
|  |  | <45 | 63/70 |  | 0.95(0.53,1.70) | 0.8542 |  |
|  |  | ≥45 | 47/40 |  | 0.56(0.36,0.85) | 0.0070 |  |
|  | BMI (kg/m^2^) | |  |  |  |  | 0.3103 |
|  |  | <30 | 71/78 |  | 0.76(0.50,1.16) | 0.2105 |  |
|  |  | ≥30 | 39/32 |  | 0.52(0.28,0.96) | 0.0359 |  |
| **Chest Wheeze** | | |  |  |  |  |  |
|  | All participants | | 110/110 |  | 0.62(0.34,1.12) | 0.1112 |  |
|  | Age (years) | |  |  |  |  | 0.9293 |
|  |  | <45 | 63/70 |  | 0.53(0.21,1.30) | 0.1642 |  |
|  |  | ≥45 | 47/40 |  | 0.56(0.24,1.29) | 0.1704 |  |
|  | BMI (kg/m^2^) | |  |  |  |  | 0.0430 |
|  |  | <30 | 71/78 |  | 0.93(0.42,2.08) | 0.8641 |  |
|  |  | ≥30 | 39/32 |  | 0.24(0.09,0.68) | 0.0068 |  |

| Subgroup | | | **n** |  | **IRR (95% CI)** | ***p* value** | ***p* value for interaction** |
| --- | --- | --- | --- | --- | --- | --- | --- |
| **Blocked Nose** | | |  |  |  |  |  |
|  | All participants | | 110/110 |  | 0.72(0.55,0.95) | 0.0182 |  |
|  | Age (years) | |  |  |  |  | 0.7621 |
|  |  | <45 | 63/70 |  | 0.71(0.48,1.07) | 0.1064 |  |
|  |  | ≥45 | 47/40 |  | 0.66(0.45,0.95) | 0.0274 |  |
|  | BMI (kg/m^2^) | |  |  |  |  | 0.5342 |
|  |  | <30 | 71/78 |  | 0.72(0.53,0.99) | 0.0453 |  |
|  |  | ≥30 | 39/32 |  | 0.59(0.33,1.04) | 0.0676 |  |
| **Runny nose** | | |  |  |  |  |  |
|  | All participants | | 110/110 |  | 0.89(0.67,1.18) | 0.4270 |  |
|  | Age (years) | |  |  |  |  | 0.0017 |
|  |  | <45 | 63/70 |  | 1.59(0.98,2.57) | 0.0604 |  |
|  |  | ≥45 | 47/40 |  | 0.60(0.41,0.87) | 0.0073 |  |
|  | BMI (kg/m^2^) | |  |  |  |  | 0.0700 |
|  |  | <30 | 71/78 |  | 1.03(0.73,1.45) | 0.8641 |  |
|  |  | ≥30 | 39/32 |  | 0.57(0.33,0.98) | 0.0421 |  |
| **Sneeze** | | |  |  |  |  |  |
|  | All participants | | 110/110 |  | 0.54(0.44,0.67) | <.0001 |  |
|  | Age (years) | |  |  |  |  | <.0001 |
|  |  | <45 | 63/70 |  | 1.76(1.22,2.53) | 0.0024 |  |
|  |  | ≥45 | 47/40 |  | 0.31(0.23,0.42) | <.0001 |  |
|  | BMI (kg/m^2^) | |  |  |  |  | <.0001 |
|  |  | <30 | 71/78 |  | 0.90(0.70,1.15) | 0.403 |  |
|  |  | ≥30 | 39/32 |  | 0.22(0.14,0.33) | <.0001 |  |
| **Earache** | | |  |  |  |  |  |
|  | All participants | | 110/110 |  | 0.48(0.27,0.88) | 0.0173 |  |
|  | Age (years) | |  |  |  |  | 0.4725 |
|  |  | <45 | 63/70 |  | 0.66(0.25,1.77) | 0.4132 |  |
|  |  | ≥45 | 47/40 |  | 0.42(0.19,0.91) | 0.0288 |  |
|  | BMI (kg/m^2^) | |  |  |  |  | 0.0540 |
|  |  | <30 | 71/78 |  | 0.31(0.14,0.70) | 0.005 |  |
|  |  | ≥30 | 39/32 |  | 1.18(0.41,3.40) | 0.762 |  |
| **Itchy Eyes** | | |  |  |  |  |  |
|  | All participants | | 110/110 |  | 0.80(0.52,1.24) | 0.3219 |  |
|  | Age (years) | |  |  |  |  | 0.8666 |
|  |  | <45 | 63/70 |  | 0.70(0.26,1.92) | 0.4920 |  |
|  |  | ≥45 | 47/40 |  | 0.77(0.47,1.26) | 0.3063 |  |
|  | BMI (kg/m^2^) | |  |  |  |  | 0.0179 |
|  |  | <30 | 71/78 |  | 0.55(0.32,0.95) | 0.0318 |  |
|  |  | ≥30 | 39/32 |  | 2.10(0.81,5.41) | 0.1255 |  |

**B.**

**B.**

**Supplementary Figure S1. The influence of age and BMI on the Lab4P-mediated reduction of (A) main URTI symptoms and (B) other potential URTI-related symptoms during the PROMAGEN study.** Statistical analysis was performed using a generalized linear model with Poisson distribution and log link function. Data are presented as the incidence rate ratio (IRR) between probiotics and placebo ± 95% confidence intervals (CI); arrows indicate that the limits of the CI is not shown. n, number of participants.

**Supplementary Figure S2. Comparison of reported URTI symptoms in summer *versus* winter.** ‘Summer’ months were defined as the first three months of the study (July – September), while ‘winter’ months were the latter three months (October – December). Comparison made using Wilcoxon rank sum test. *: *p*<0.05.

# References:

1. Michael DR, Jack AA, Masetti G, Davies TS, Loxley KE, Kerry-Smith J, et al. A randomised controlled study shows supplementation of overweight and obese adults with lactobacilli and bifidobacteria reduces bodyweight and improves well-being. Sci Rep 2020; 10:4183.

2. Bolyen E, Rideout JR, Dillon MR, Bokulich NA, Abnet CC, Al-Ghalith GA, et al. Reproducible, interactive, scalable and extensible microbiome data science using QIIME 2. Nat Biotechnol 2019; 37:852-7.

3. McMurdie PJ, Holmes S. phyloseq: an R package for reproducible interactive analysis and graphics of microbiome census data. PLoS One 2013; 8:e61217.

4. Oksanen J, Blanchet FG, Kindt R, Legendre P, Minchin PR, O'Hara RB, et al. vegan: Community Ecology Package. 2013.

5. Callahan BJ, McMurdie PJ, Rosen MJ, Han AW, Johnson AJ, Holmes SP. DADA2: High-resolution sample inference from Illumina amplicon data. Nat Methods 2016; 13:581-3.
